# Cost effectiveness of a controlled lifestyle intervention for pregnant women with obesity

**DOI:** 10.1186/s12884-021-04098-5

**Published:** 2021-09-21

**Authors:** Hanna Gyllensten, Karin Haby, Marie Berg, Åsa Premberg

**Affiliations:** 1 Institute of Health and Care Sciences, University of Gothenburg, Box 457, SE-405 30, Göteborg, Sweden; 2 Centre for Person-Centred Care - GPCC, University of Gothenburg, Gothenburg, Sweden; 3 Department of Clinical Neuroscience, Karolinska Institutet, Stockholm, Sweden; 4 Region Västra Götaland, Research and Development Primary Health Care, Gothenburg, Sweden; 5 Region Västra Götaland, Sahlgrenska University Hospital, Department of Obstetrics, Gothenburg, Sweden

**Keywords:** Obesity, Pregnancy, Maternal health services, Diet, Food, And nutrition, Physical activity, Economic evaluation, Gestational weight gain

## Abstract

**Background:**

The Mighty Mums antenatal lifestyle intervention is a person-centered behavioral intervention focusing on nutrition and physical activity for pregnant women with obesity (body mass index [BMI] ≥30). The aim of this study was to evaluate the costs and clinical outcomes of adding the Mighty Mums intervention to standard antenatal care.

**Methods:**

Participants in the intervention group (*n* = 434) received motivational talks with their midwife and a selection of physical and/or nutritional activities in addition to antenatal care. Control participants (*n* = 867) from adjacent geographic areas received standard antenatal care. Costs for staff, unit costs for specific activities, and registered costs for specialized antenatal care were analyzed for associations with gestational weight gain and self-reported health. Results are reported for the intention-to-treat (ITT) population and a per protocol (PP) population identified by participation in the intervention. Analyses included bootstrapped linear regressions adjusted for background characteristics that differed significantly between groups.

**Results:**

The average costs were SEK 9727 higher (95% confidence interval [CI]: 6677 to 12,777) among participants in the intervention group than in the control ITT population and SEK 8655 (95% CI 4586 to 12,724) higher than in the PP population. The cost increase per 1 kg reduction in gestational weight gain was SEK 12,369 in the ITT population and SEK 7209 for the PP population.

**Conclusion:**

Participation in the Mighty Mums intervention was associated with higher costs, but also reduced gestational weight gain. The cost per kilogram reduction in gestational weight gain was low, particularly in the PP population. A future decision to implement this behavioral intervention in standard care should take into account society’s willingness to pay per unit reduction in gestational weight gain.

**Trial registration:**

The study is registered at ClinicalTrials.gov, Identifier: NCT03147079.

**Supplementary Information:**

The online version contains supplementary material available at 10.1186/s12884-021-04098-5.

## Background

Maternal obesity is associated with both increased risk of complications for mothers and children [1] and increased healthcare costs [2, 3] Women with obesity have higher risks than other women for gestational diabetes, pre-eclampsia, gestational hypertension, depression, instrumental or cesarean birth, preterm birth, large-for-gestational-age babies, fetal defects, congenital anomalies, and perinatal death, lower rates of breastfeeding initiation, and greater risk of early breastfeeding cessation [4]. A recent literature review reported that the incremental costs (i.e., the difference in costs between those with and without the condition) of obesity during pregnancy ranges from EUR 191 to EUR 16,046, indicating large differences in costs between countries and the conditions included in individual studies [5].

In addition to complications during pregnancy, children of mothers who were obese before pregnancy are at increased risk of all-cause hospitalizations during their first 5 years [6]. Chronic diseases such as cardiovascular disease, metabolic syndrome, and type 2 diabetes later in the child’s life have been associated with the mother’s obesity during pregnancy [1]. In Europe, it is possible to distinguish a gradient in the prevalence of obesity in children under 10 years, which varies from 10% in the north of Europe to up to 40% in the south [7]. Risks associated with obesity in children span a wide range of biological, social, and environmental factors [8], including regional factors such as human development index and average income in the area of residence [9]. Among the main risk factors for obesity in children is parental BMI [10], and an additive effect of obesity and diabetes during pregnancy has been reported in both maternal health outcomes during pregnancy and child health after birth [11].

Knowledge of effective interventions to reduce the negative effects of obesity during pregnancy is insufficient [12]. Previous bariatric surgery has been shown to reduce a number of complications in obese mothers during pregnancy, such as gestational diabetes, hypertensive disorders, and macrosomia [13], but it is also associated with surgical and internal adverse outcomes (including nutrient deficiency) during pregnancy [12]. Supervised exercise and counseling about home exercise has also shown positive results in overweight women [14]. Overall, behavioral interventions, particularly diet-based interventions [15], appear to be effective in reducing gestational weight gain in obese women during pregnancy [16, 17]. Variations in tested interventions make comparisons difficult [18], but interventions to reduce gestational weight gain have been reported to reduce costs and adverse health outcomes [19].

We previously demonstrated that the Mighty Mums person-centered behavioral intervention decreased gestational weight gain among pregnant women with obesity [20]. Person-centered care has been suggested as a way to involve patients in their own health decisions [21], which may be particularly important in primary healthcare, including antenatal care and lifestyle interventions. Healthcare decision-making should, however, also consider the cost effectiveness of new programs, since resources are scarce [22]. The aim of this study was to evaluate the costs associated with the clinical outcomes of including the Mighty Mums intervention in standard antenatal care.

## Methods

### Study population

We conducted a controlled trial in 2011 to 2013 to evaluate the Mighty Mums intervention, a behavioral antenatal care intervention aimed at pregnant women with BMI ≥30, in Gothenburg, Sweden [20, 23].

The intervention group (*n* = 459) was recruited from antenatal care services in the Gothenburg area, with a source population of approximately 2500 pregnant women with BMI ≥30 during the study period. A group of internal controls (*n* = 105) were recruited from a source population of approximately 800 in other antenatal care units in the same geographic area. Women eligible for participation, in either the intervention or internal control groups, were informed about the study and asked for consent to participate by their midwife during their first visit to the antenatal care. To avoid biased results from only highly motivated women participating in either the intervention or the internal control group, the intervention was delivered through the standard antenatal care system. After informed consent, participants were followed during pregnancy and until postpartum checkup through register data and data collected specifically for the study. Women were excluded from the analyses if the pregnancy ended in abortion or miscarriage or if the first antenatal care visit occurred later than week 20. An external control group (*n* = 790) was identified in an adjacent geographic area and followed using register data covering all pregnant women of BMI ≥30 in the register from that area. Register data were retrieved from the Swedish Maternity Health Care Register [24]. An overview of the inclusion process is presented in the study flow diagram (Additional file 1: Fig. S1).

Participation in other parallel interventions was a reason for exclusion from the intervention and internal control groups, but we had no information about such participation in the external controls.

### The antenatal care and the behavioral intervention

All participants (the intervention group, internal controls, and external controls) received standard antenatal care which included midwife care during pregnancy and postpartum visits 2 to 3 months after childbirth—usually a total of 11 to 12 visits [25, 26]. In Sweden, antenatal care is midwife-driven, provided free of charge to all pregnant women and includes regular health checks, screening, psychological support, and education [27].

The intervention involved midwives using motivational interviewing [28] and person-centered care [21]. Women in the intervention group were scheduled to receive two extra appointments with the midwife. Motivational talks, personalized counselling on food and physical activity were added to the visit to the midwife, as well as follow-up on the woman’s goals and progress throughout the pregnancy. They were also offered participation in activities (sub-interventions) directed toward nutrition and physical activity and able to use these according to their personal choices. Sub-interventions included visits with a dietician, individually or in group, aqua aerobics, walking poles, and pedometers as well as prescribed physical activity and information about lifestyle activities in community health centers. The information on physical activity and healthy eating was delivered by the midwife during the extra appointments at the beginning of the pregnancy, and about 5 min of each appointment during the pregnancy were dedicated to lifestyle follow-up and support. Adherence was monitored until postpartum checkup in a logbook designed for the Mighty Mums Project. Specific information about prescribed physical activities, associated contacts, and/or the use of community health centers was not available for the analyses. The women in the intervention group were encouraged to gain less than 7 kg during pregnancy.

### Health outcomes

The primary health outcome measure of the study was gestational weight gain from enrollment to last pregnancy visit (weight reported to the Maternity Health Care Register). Change in self-rated health between enrollment and postpartum checkup was also reported to provide a broader perspective of health effects of the intervention.

### Costs

The cost calculation was prevalence-based and included costs from enrolment to postpartum checkup for each participant (i.e., approximately 9 months), covering the relevant costs and the health outcomes. Studied costs included antenatal care visits, intervention participants’ personalized use of sub-interventions, tests related to suspected complications, specialized antenatal care for occurring complications, and childbirth. Childbirth costs were calculated based on Robson groups, a classification system for births that is based on maternal and childbirth characteristics [29], and for which there is regional cost statistics available [30]. Interpreter costs were reported separately since a high number of intervention participants used interpreter services [20], which should be viewed more as an indicator of the broad recruitment rather than a cost resulting from the intervention. Costs for each resource are described in Table 1. Because of the short time horizon, no discounting was conducted. Costs were adjusted for inflation using a national inflation factor for healthcare wages, excluding costs for drugs [32].

**Table 1:** Resource use and unit costs for each resource for intervention and control groups

Resource	Unit	Price	ITT population		PP population	
			Intervention	Control	Intervention	Control
		SEK	*N* = 434	*N* = 867	*N* = 115	*N* = 841
antenatal care visits						
Midwife, first two visits	45 min	345/h^a^	868 visits	1734 visits	230 visits	1682 visits
Midwife, subsequent visits	30 min	345/h^a^	4159 visits (mean: 9.6)	8201 visits (mean: 9.5)	1195 visits (mean: 10.4)	8024 visits (mean: 9.5)
Midwife, post-natal visit	30 min	345/h^a^	362 visits	767 visits	107 visits	755 visits
Physician	NA					
Interpreter		550/h + 100/visit [31]	45 women/557 visits	17 women/199 visits	14 women/183 visits	17 women/199 visits
sub-interventions						
Individualized dietary advice from a dietitian	60 min	310/h^a^	69 women/83 visits	20 women/31 visits	16 women/18 visits	16 women/24 visits
Food discussion groups led by a dietitian	90 min, 5 participants	310/h^a^	62 women/145 visits	NA	26 women/59 visits	NA
Walking poles	1 pair	183/pair	86 women	NA	34 women	NA
Pedometers	1 unit	60/unit	148 women	NA	45 women	NA
Information about community health centers offering lifestyle education	Not listed		Unknown	NA	Unknown	NA
Aqua aerobics	60 min (mean 4.86 participants)	1000^b^ per maximum 10 participants	74 women/532 visits	NA	25 women/214 visits	NA
Midwife	60 min	345/h^a^	146 events	NA	–	NA
specialized antenatal care			54 women	99 women	9 women	98 women
Mother’s complications^c^	mother	From ELVIS^d^	53 (12.2%)	103 (11.9%)	9 (7.8%)	102 (12.1%)
Childbirth	mother	Published^e^	434	867	115	841
Child’s complications	child	Unknown	91 (21.0%)	177 (20.4%)	21 (18.3%)	163 (19.4%)

*SEK* Swedish krona, *ITT* Intention-to-treat, *PP* Per protocol.

^a^Average wage, including costs for professional development and continuing education.

^b^Template cost varied somewhat during the study period based on number of participants and fees.

^c^Includes gestational diabetes, gestational hypertension, and pre-eclampsia.

^d^Individual-level information collected from the administrative registers (ELVIS) at the local specialized antenatal care unit.

^e^Mean cost by Robson group, identified using information about Cesarean section and parity [30].

Complications in mothers resulting in specialized antenatal care included decreased glucose tolerance, gestational diabetes, gestational hypertension, and pre-eclampsia. For women with specialized antenatal care, costs were retrieved from an administrative register at the hospital (ELVIS register). Register costs were assumed to include the costs for childbirth if inpatient care costs were SEK 20,000 or more. A sensitivity analysis was conducted in which inpatient costs of SEK 15,000 or SEK 10,000 were also assumed to include costs during childbirth to add more conservative estimates for specialized healthcare costs.

Costs were not calculated for routine encounters and tests conducted by assistant nurses, as these were not registered in all antenatal care units. However, these services were assumed to be used to the same extent by participants in the intervention and standard care groups. The same applies for physician visits in antenatal care units.

Costs for conducting the controlled trial were reported separately, including costs for designing the study and educating participating midwifes and other antenatal healthcare professionals (based on 4 h each at a mean wage of SEK 345/h for 80 educators and participants).

### Analyses

Imputation analyses using fully conditional specifications were conducted for missing data on postpartum weight, self-reported health, and antenatal care visits (seed for imputation: 4918 [20];. Predictive means matching was used for the imputation of antenatal care visits. The covariates included in the imputation of postpartum weight and self-reported health were baseline and postpartum measures, height, parity, age at enrolment, country of birth, use of interpreter, education, occupation, and use of nicotine [20]. The imputation of antenatal care visits included the numbers of visits registered in Swedish Maternity Health Care Register, numbers of postpartum visits, parity, pregnancy weeks, number of weeks past 40 weeks at birth, weight at enrollment (transformed to week 15 weight if enrollment occurred after week 15 using a national algorithm [33];, occupation, the mother’s country of birth, and use of interpreter services.

Analyses were conducted both for the total study population and for a subset reported to have received the intervention PP [20], including at least three (out of seven) notes in the logbook of at least partly completed nutritional and physical activities. Maximum attendance in the intervention would imply seven visits with the midwife (i.e., seven occasions when the pregnant woman and her midwife discussed the intervention) and high adherence to the planned participation. Reporting both intention-to-treat results, including all included participants, and PP analyses selecting out those actually participating at a high level, allows better understanding of the effectiveness and efficacy of the intervention [34]. Moreover, PP participation required at least to have contributed with height and weight at enrollment, and weight at the end of pregnancy (thus, no imputation of the primary outcome was necessary).

The average costs per person (i.e., per pregnancy) were calculated from the resources used by each person, including antenatal care and tests for suspected complications, the intervention, and care for complications. Costs were reported as total average cost per person and by sociodemographic factors (age and BMI at enrolment, education, occupation, and smoking before pregnancy). All between-group differences, proportions, and means were tested for statistical significance (*P* < 0.05). Because of the skew in the data, confidence intervals (CIs) were calculated using a bootstrap methodology [35], and differences were thus calculated using regression analyses with bootstrap. All adjusted analyses were conducted using country of birth, use of interpreter services, occupation, and BMI at enrollment as covariates.

The incremental cost-effectiveness ratio was calculated as the bootstrapped ratio of the difference in average total costs (excluding interpreter services) between intervention and control groups divided by the difference in gestational weight gain between the groups [36], thus indicating the cost for decreasing the gestational weight gain by 1 kilogram. As a sensitivity analysis, bootstrapped differences in costs and decreases in gestational weight gain were presented in a cost-effectiveness plane. Additionally, changes in self-reported health (Likert scale) were reported. ANOVA was used to examine the effect of intervention group and self-reported health at enrollment and postpartum. Ordered logistic regression was used to examine the effect of intervention group and self-reported health at enrollment on self-reported health postpartum, without and with adjustment for covariates. A sensitivity analysis of the cost distribution was conducted in the form of a tornado diagram, by calculating the mean total costs among groups of participants in the intervention group based on the sub-interventions used, according to each individual’s choices.

Analyses were conducted in Stata/SE 16.1 (StataCorp).

Ethical approval for the study was received from the Regional Ethical Review Board in Gothenburg (#505–10) in accordance with Helsinki declaration. The study has been registered at ClinicalTrials.gov, identifier: NCT03147079. 10/05/2017.

## Results

### Characteristics of the study population

A larger proportion of women in the intervention group had a BMI ≥ 40 while a larger proportion of women in the control group were in the 30–35 BMI range (only in the ITT population) and/or were employed (Additional file 1: Table S1). As previously reported [20], it was also more common for women in the intervention group to be born in another country and to use interpreter services. Differences in the distribution of complications between groups were also reported (Additional file 1: Table S1).

The proportions of women with complications expected to result in specialized antenatal care were similar in the intervention and control groups (intervention: 54 [12%]; control: 99 [11%]). However, for 383 (88%) of intervention group participants, and 814 (94%) of controls, the hospital register did not include any specialized antenatal care. This indicates control group participants were to a larger extent not visiting the specific hospitals, from which data was available in this study, during complications.

### Cost outcomes

After adjusting for background characteristics that differed significantly between groups, average costs were SEK 9727 (95% CI: 6677 to 12,777; unadjusted: SEK 10,385, 95% CI: 7490 to 13,280) higher among participants in the intervention group than in the control group (Table 2). Including interpreter services resulted in similar results (adjusted: SEK 9717; 95% CI: 6667 to 12,767; unadjusted: SEK 9966; 95% CI: 7088 to 12,845) in the ITT comparison. The unadjusted mean difference in costs for specialized antenatal care was SEK 7101 (95% CI: 4163 to 10,038), with higher costs for the intervention group. Limiting the analyses to participants adhering to the protocol (PP), we found that the cost difference was SEK 8953 (95% CI: 4915 to 12,991) or SEK 8655 (95% CI: 4586 to 12,724) after adjustment (Table 2).

**Table 2:** Average costs (SEK) by cost components for different population groups and the differences between groups

Background characteristics	ITT population			PP population		
	Intervention	Controls	Difference	Intervention	Controls	Difference
	*Mean* (95% CI)	*Mean* (95% CI)	Unadjusted *M* (95% CI) Adjusted *M* (95% CI)†	*Mean* (95% CI)	*Mean* (95% CI)	Unadjusted *M* (95% CI*)* Adjusted *M* (95% CI)^a^
**Total cost, including interpreter services**	**43,691 (41,073 to 46,309)**	**33,306 (32,180 to 34,433)**	**10,385 (7490 to 13,280)** **9727 (6677 to 12,777)**	**42,707 (38,823 to 46,590)**	**33,218 (32,109 to 34,327)**	**9489 (5450 to 13,528)** **8693 (4627 to 12,758)**
Interpreter	510 (362 to 658)	91 (47 to 136)	418 (265 to 572) 10 (−13 to 33)	630 (329 to 932)	94 (50 to 139)	536 (222 to 850) 38 (−9 to 85)
**Total cost, excluding interpreter services**	**43,181 (40,578 to 45,784)**	**33,215 (32,089 to 34,341)**	**9966 (7088 to 12,845)** **9717 (6667 to 12,767)**	**42,076 (38,206 to 45,947)**	**33,124 (32,018 to 34,229)**	**8953 (4915 to 12,991)** **8655 (4586 to 12,724)**
Antenatal and childbirth costs						
Antenatal midwife visits	2314 (2261 to 2367)	2302 (2265 to 2339)	13 (− 50 to 76) 30 (− 35 to 95)	2471 (2383 to 2558)	2318 (2281 to 2356)	152 (51 to 253) 176 (75 to 277)
Hospital-based care	34,333 (31,862 to 36,804)	30,157 (29,078 to 31,253)	4177 (1447 to 6907) 4784 (1866 to 7703)	32,296 (28,719 to 35,873)	30,180 (29,107 to 31,253)	2116 (− 1666 to 5897) 2348 (− 1438 to 6134)
Specialized antenatal care^b^	9415 (6552 to 12,278)	2314 (1570 to 3058)	7101 (4163 to 10,038) 7706 (4583 to 10,829)	4821 (899–8742)	2356 (1624 to 3087)	2465 (−1180to − 6110) 2874 (− 800 to − 6548)
Childbirth^b^	24,918 (23,561 to 26,276)	27,842 (26,893 to 28,791)	− 2924 (4587 to − 1261) − 2922 (− 4653 to − 1190)	27,475 (24,885 to 30,066)	27,825 (26,913 to 28,736)	− 349 (− 3088 to 2389) − 546 (− 3436 to 2384)
Intervention costs						
Food discussion groups led by a dietitian	1864 (1420 to 2309)	0	1864 (1392 to 2336) 2114 (1584 to 2645)	2863 (1788 to 3938)	0	2863 (1803 to 3923) 3079 (1967 to 4190)
Individualized dietary advice from a dietitian	3557 (2686 to 4428)	665 (327 to 1004)	2892 (1986 to 3798) 2098 (1283 to 2914)	2911 (1441 to 4382)	531 (243 to 819)	2381 (869 to 3892) 2051 (689 to 3412)
Pedometers	20 (18 to 23)	0	20 (18 to 23) 20 (18 to 23)	23 (18 to 29)	0	23 (18 to 29) 23 (18 to 29)
Walking poles	36 (29 to 43)	0	36 (30 to 43) 37 (30 to 44)	54 (39 to 69)	0	54 (39 to 69) 55 (39 to 70)
Aqua aerobics	545 (401 to 690)	0	545 (398 to 693) 622 (456 to 788)	828 (483 to 1173)	0	828 (478 to 1177) 886 (522 to 1249)

*CI* Confidence interval, *ITT* Intention-to-treat, *PP* Per protocol, *SEK* Swedish krona.

Figures are rounded.

^a^Adjusted for country of birth, use of interpreter services, occupation, and BMI at enrolment.

^b^For 63 participants, specialized antenatal care records included inpatient care over SEK 20,000, which was assumed to include costs for childbirth and thus were not included as template costs based on Robson group.

Mean intervention costs (ITT) were SEK 5359 (95% CI: 4406 to 6311). However, this varied from SEK 688 (95% CI: − 4093 to 5469) among those with BM I ≥ 40 to SEK 6644 (95% CI: 4316 to 8972) among with BMI 35–40 at enrollment (Additional file 1: Table S2).

Educating participating midwifes and other antenatal care healthcare professionals resulted in additional costs of SEK 110,400 for conducting the Mighty Mums intervention.

### Health outcomes

As previously reported [20], the Mighty Mums intervention program resulted in a lower gestational weight gain if comparing participants in the intervention group to those in the control group, using the ITT population (unadjusted difference: − 0.9 [95%CI: − 1.7 to − 0.2] kg; adjusted difference: − 0.2 [95% CI: − 1.0 to 0.6] kg). Corresponding results for the PP population were unadjusted difference: − 2.3 (95% CI: − 3.5 to − 1.2) kg, and adjusted difference: − 1.5 (95% CI: − 2.6 to − 0.3) kg (Additional file 1: Table S2). Self-reported health is reported in Additional file 1: Fig. S2. Self-reported postpartum health was higher among controls in unadjusted analyses for the ITT population (*P* = 0.024), but not in the adjusted analyses (*P* = 0.073) or in the PP analyses (*P* = 0.299 unadjusted and *P* = 0.402 adjusted).

### Cost effectiveness

The incremental cost-effectiveness ratio was SEK 11,004 per 1 kg reduction in gestational weight gain in the ITT population. The corresponding result for the PP population was SEK 3841.

### Sensitivity analyses

**Fig. 1:**
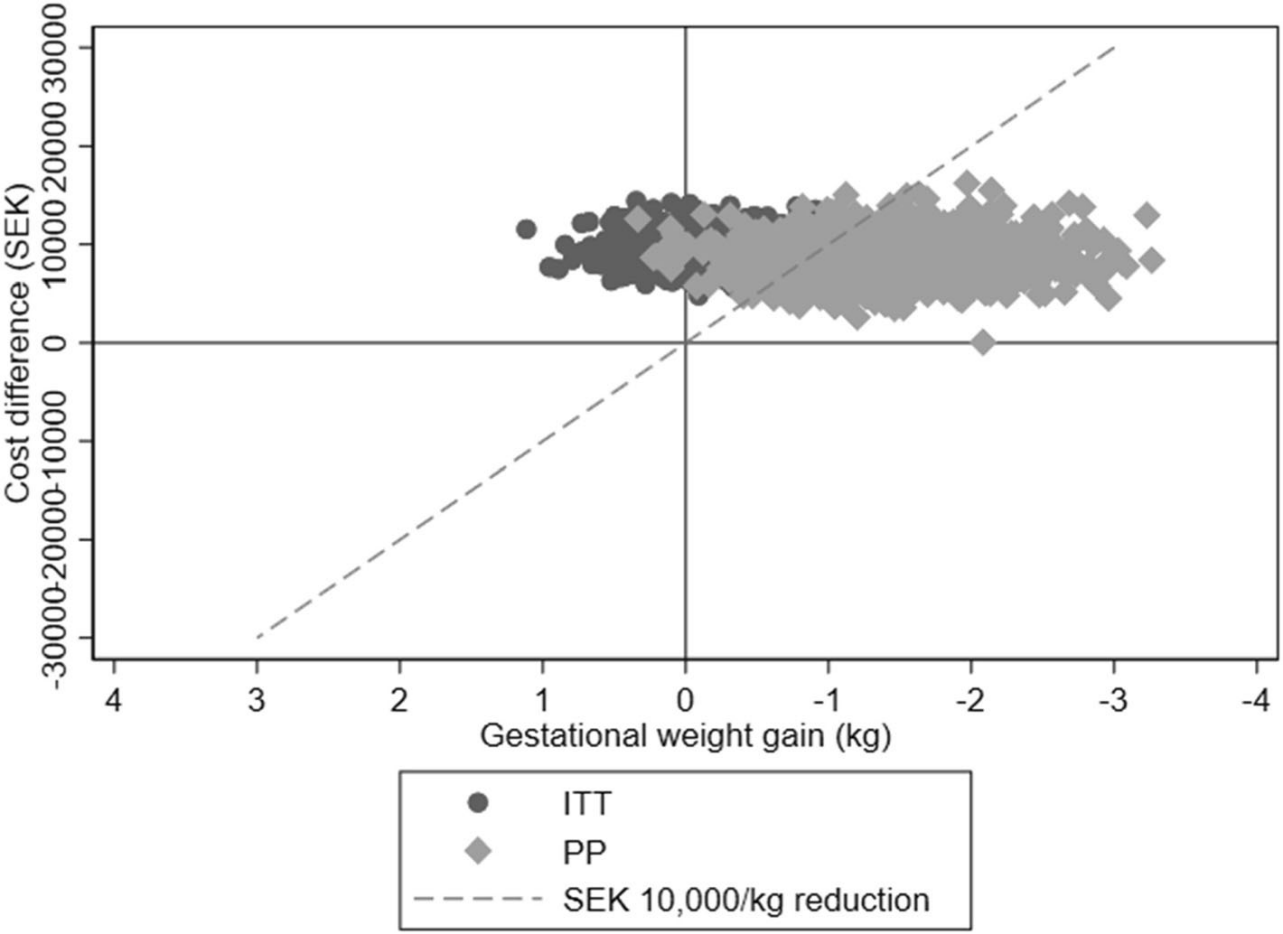
Incremental cost-effectiveness plane of the Mighty Mums intervention (adjusted analyses). ITT = intention to treat; PP = per protocol; SEK = Swedish krona. The x-axis is inverted to ensure an assumed positive health outcome (i.e., reduced gestational weight gain) is shown to the right in the figure

The cost-effectiveness plane is shown in Fig. 1, with a hypothetical threshold value of SEK 10,000/kg reduction in gestational weight gain indicated by a dashed line.

**Fig. 2:**
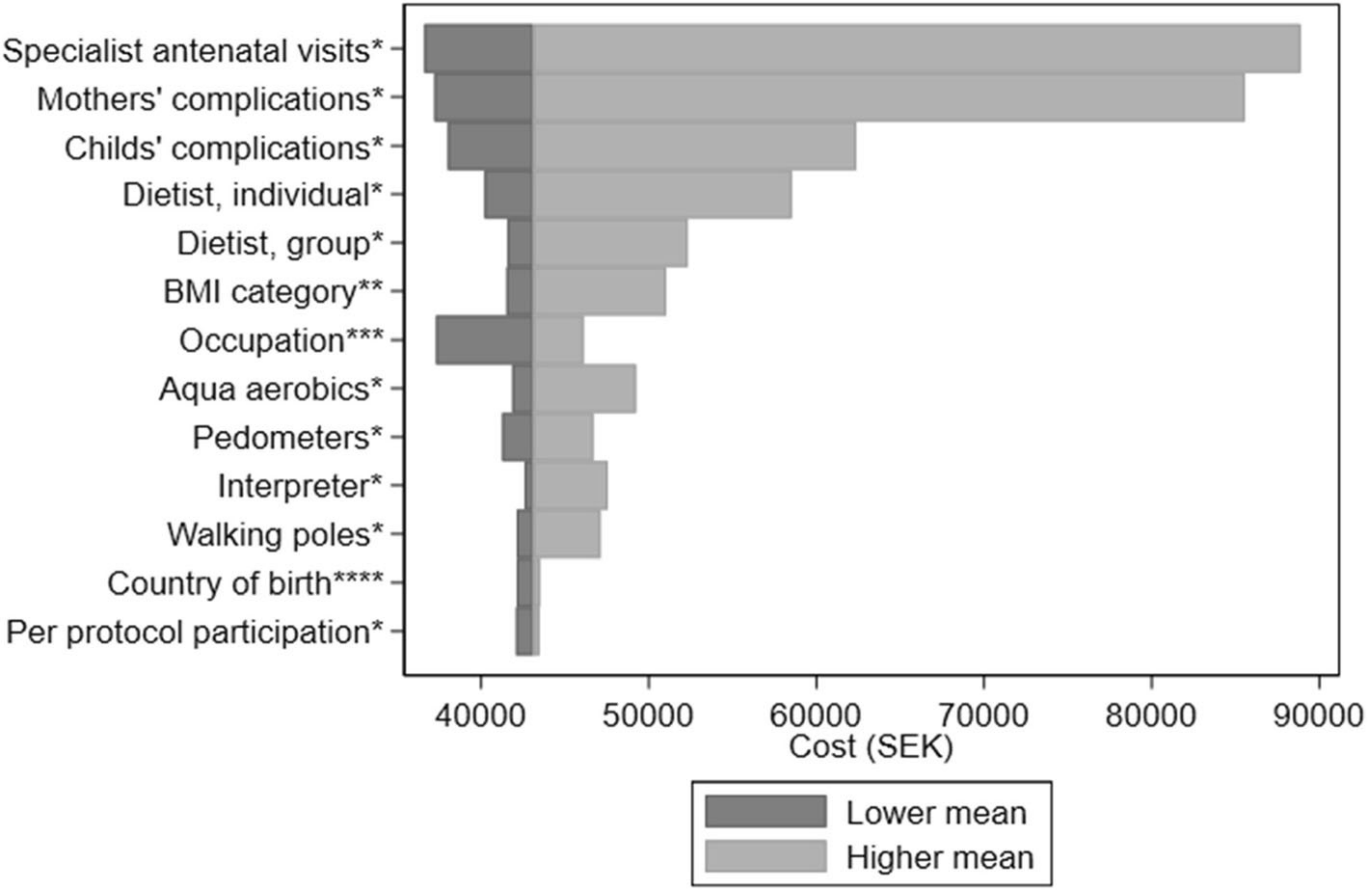
Univariate analysis of the distribution of mean costs (excluding interpreter services) by health outcomes and background characteristics of participants in the Mighty Mums intervention group. BMI = body mass index; SEK = Swedish krona. Categories indicated by lower mean v. higher mean: * No v. yes. ** BMI 30–35 v. BMI ≥ 40. *** Other v. paid work. **** Sweden v. other

According to the one-way sensitivity analysis, the cost outcomes among participants in the intervention group were more sensitive to adverse pregnancy outcomes and the background characteristics of the participants than those directly affected by the intervention (Fig. 2).

**Fig. 3:**
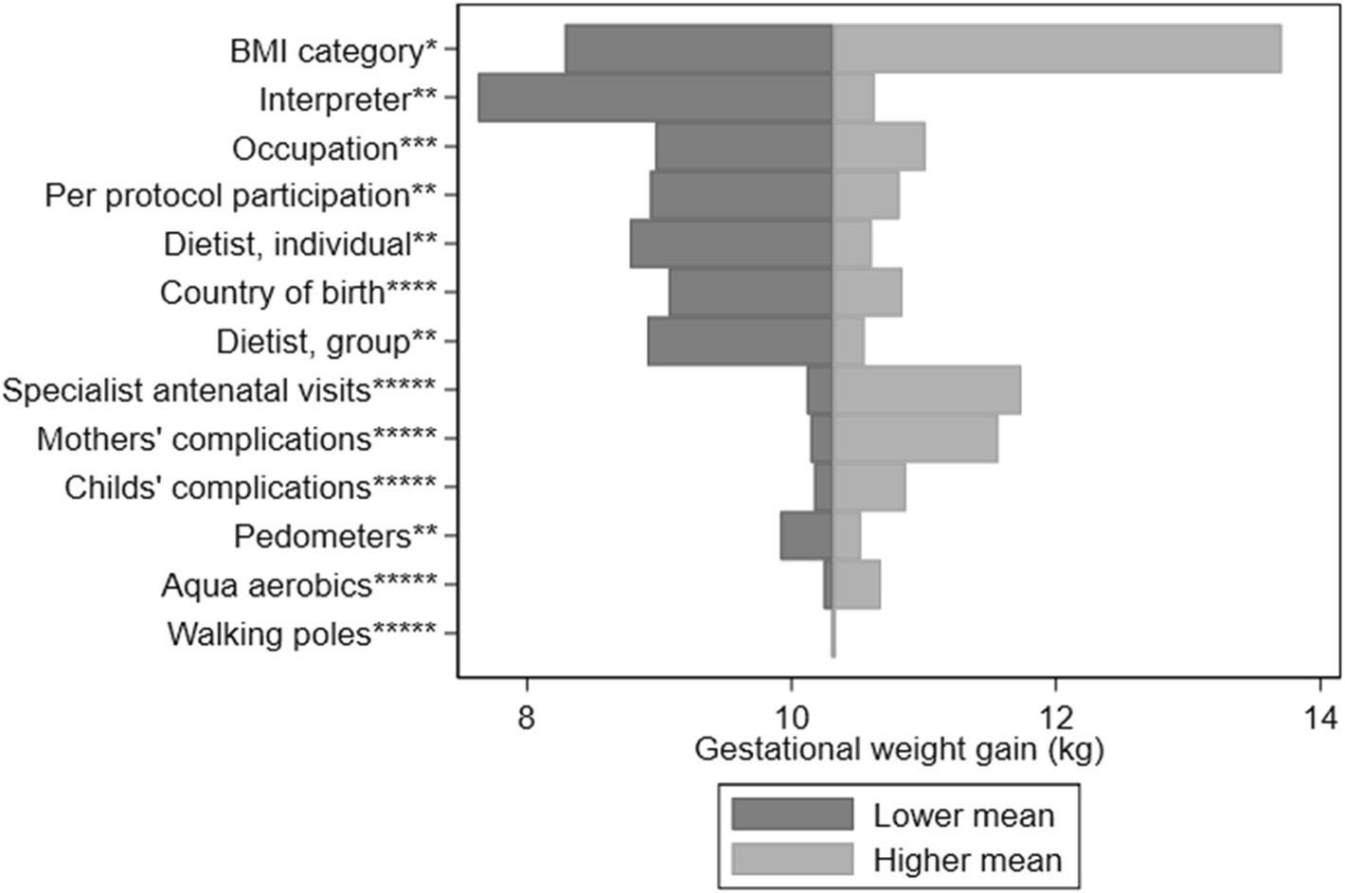
Univariate analysis on the distribution of mean gestational weight gain by health outcomes and background characteristics of participants in the Mighty Mums intervention group. BMI = body mass index; kg = kilograms. Categories indicated by lower mean v. higher mean: * BMI ≥ 40 v. BMI < 30. ** Yes v. no. *** Other v. paid work. **** Sweden v. other. ***** No v. yes

Conversely, gestational weight gain (Fig. 3) was mainly sensitive to BMI categories. No statistically significant effect of self-reported health was found for costs (*P* > 0.3 in both ITT and PP analyses).

## Discussion

Healthcare costs were approximately SEK 10,000 higher among women receiving the Mighty Mums intervention as an add-on to antenatal care than among women receiving standard antenatal care. The cost per kilogram reduction in gestational weight gain was SEK 11,004, but the cost was considerably lower if only the PP population was analyzed (SEK 3841). These estimates correspond to (unadjusted/adjusted mean values) EUR 907/EUR 884 higher overall costs for participants in the intervention, and a corresponding incremental cost-effectiveness ratio of EUR 1001 per kilogram reduction in gestational weight gain in the ITT population, and EUR 349 in the PP population, inflated to 2018 values [32] and converted to EUR using purchasing power parities [37]. However, differences in background characteristics between the groups, such as BMI at enrollment appear to have affected health outcomes. Moreover, costs appear to have been mainly sensitive to complications. Limitations in the data collection make it unfeasible do draw conclusive results regarding the costs of complications.

Major strengths of the study were the population-based approach and delivery through standard antenatal care, which could allow the effects to be identified as relevant and applicable to standard antenatal care. The main limitation, however, was the lack of randomization and low recruitment of participants for the internal control group, which negatively affects the generalizability of results. Although the sociodemographic background in the studied geographic areas were expected to be similar, the resulting intervention group included an unexpectedly higher proportion of women needing interpreter services than in women in the control group. In the analyses, the costs for interpreter services were thus reported separately. In addition to causing extra costs in antenatal care, such differences in background characteristics could potentially result in additional costs for healthcare overall, which was not possible to derive using the retrospectively collected data used in this study. It has previously been reported that immigrant women in Sweden have a higher risk of severe maternal morbidity [38], which has been partially explained by their higher likelihood to receive sub-optimal and negative care experiences [39]. The low recruitment may also have resulted in a selection of participants, especially in the internal control group, based on the midwives’ and/or the women’s own motivations to participate. Concerns have been raised about how well the effect of behavioral interventions can be studied, as mothers choosing to enter a lifestyle intervention are likely to be more motivated to make changes to their lifestyle during pregnancy regardless of their randomization into the intervention or the control group [40]. Additionally, 20 women (20%) of those in the internal control group (most with BMI > 40) received individualized dietary advice as part of their antenatal care, allowing the argument that these women received part of the intervention. In fact, these selected women did receive, on average, more encounters with the dietician than participants in the intervention (1.6 visits vs. 1.2 for those using this specific resource). Underpowering is common in so called “piggyback” economic evaluations, studies where the economic evaluation is added in a later stage as an add-on to the main study design, that sample sizes based on the clinical evaluation [41]. This mean that the sample size is not large enough to reject the null hypothesis of no difference between the groups. However, in addition to underpowering, the analyses in this study are also hindered by the quasi-experimental design and the apparently biased recruitment.

One limitation of our cost estimations was our use of Robson categories. Since no information was registered on breech presentation or induction of birth, the analyses could only take into account some of the factors affecting Robson group [29]. We thus underestimated the total costs of births in this study, since factors that could have resulted in births being listed in more costly Robson categories were unaccounted for. Additionally, when collecting data retrospectively from the hospital records, it was found that the register did not fully associate costs and specific health encounters, thus resulting in an inability to deduce whether costs for the specialized antenatal care included costs for the hospital admission during labor and delivery. In combination with apparent missing data on costs for specialized antenatal care for a group of participants in the control group, the increase in costs resulting from the intervention appears to be exaggerated. The dietitian group and individual meetings with a dietitian were the most expensive sub-interventions in this study, but the costs for these services were also highly related to the number of people participating, and costs per participant could thus be decreased through increased participation if the program were provided to a larger population.

Our results are in line with a previous meta-analysis, which found that antenatal diet and physical activity interventions reduced gestational weight gain by an average of 0.7 kg, but with no associated effects on complications [42]. A Cochrane review found similar results, but with possibly reduced risks of gestational diabetes mellitus and cesarean section [43]. We found no such reductions in our data, although the proportion with cesarean section was already lower in all studied groups (20–23%) than in previously reported results (299 per 1000 and 284 per 1000, respectively [43];. This study used a person-centered approach, and thus the woman and her midwife jointly decided the balance between diet and exercise for each participant. The PP analysis included women who fulfilled their goals for both diet and exercise, but we do not know if that means the woman actively conducted both types of interventions as they would depend on the goals set. However, previous research suggests that both diet and exercise, in isolation, and the combination, all have shown beneficial effects during pregnancy [44, 45]. Thus, it is possible that there were women judged as not fulfilling the PP level who still performed well on either diet or exercise and had beneficial effects of the intervention. Applying stricter cut-offs for inclusion in the PP analyses also resulted in a trend toward even lower gestational weight gain (unpublished results), although at the cost of reduced sample size. The corresponding effect on costs was negligible. One possible interpretation is that the intervention had the expected effect on weight, and that the comparably higher costs in the intervention group were associated not with participation in the study but rather with data limitations. Moreover, the follow-up enabled personalized feedback and discussions about the individuals’ performance and potential changes to the set goals. Individualized feedback appears to be an important factor in promoting healthy behavioral changes [46]. This study was not set up to examine long-term effects or costs. It is possible that an intervention such as this will affect behavior long-term, and thus potentially have beneficial effects on the future health of both mother and child. In our follow-up, when the children have reached 2.5 years of age (unpublished results), long-term analyses will be even more difficult, due to non-response, consecutive pregnancies, surgery, and other activities conducted to handle the obesity.

Although the Mighty Mums antenatal lifestyle intervention has demonstrated effectiveness in reducing gestational weight gain among those adhering to their set goals, limitations in data collection make conclusive estimates of its cost effectiveness challenging. Moreover, differences in characteristics and data availability between study groups make assessment of opportunity costs unfeasible. The additional cost for providing the intervention was small compared to the total cost for antenatal care in this patient group, and the cost for educating personnel and developing the intervention was about the same as caring for three pregnancies. The only components available to assess the costs of executing the intervention [47] was that of the midwives’ additional education. No further assessment was made of costs for developing the intervention. It is possible that there were additional costs attached to both developing the intervention and implementing it in antenatal care, but those were not collected or possible to deduce retrospectively. Future studies should ensure all costs associated with implementing such interventions are explored, in accordance with recommended practices [47], Based on the available data, no conclusions can be drawn on how often the educational program needs to be repeated to ensure personnel are updated. However, it should be natural to include it in in-service training to carry out the guidelines for managing overweight and obesity in pregnancy. The person-centered aspects of the intervention reflect the transition in Swedish healthcare to meet patients on a more individual basis, and most regions are now committed to developing such partnerships with patients [48]. The findings regarding the sub-interventions may be used to guide future initiatives. The costs varied between components from very small (e.g., for pedometers) to more resource intense activities such as dietician visits. However, the one-way sensitivity analyses indicate that at least some of the more resource-consuming activities (e.g., dietitian contacts and aqua aerobics) were more frequent among women who managed better to restrict their gestational weight gain.

Before introducing Mighty Mums or a similar intervention, evidence is needed of its potential beneficial effects on other aspects of care or the health of pregnant women with BMI ≥30, because current data indicate small effects on gestational weight gain and costs that are not negligible. To draw conclusions about the actual value of such an intervention, a value needs to be put on the potential health effects being measured. For example, what is the value, or how much is society willing to pay, for a specific decrease in gestational weight gain? This depends on what is the actual effect of decreasing gestational weight gain. This has also been discussed in a recent health economic evaluation of interventions to reduce gestational weight gain as part of decreasing gestational diabetes, in which the decided threshold was found to affect the cost effectiveness of said interventions [49]. The same applies to acceptable costs for reducing adverse maternal outcomes, such as gestational diabetes or hypertensive disease in pregnancy [50]. Thus, more research is needed on the long-term effects of decreasing gestational weight gain through antenatal care interventions, including their potential effects on health-related quality of life, to enable comparisons of cost effectiveness with other healthcare interventions. Moreover, future research needs to clarify whether pregnancy is a beneficial period to create change in healthy lifestyles, or if interventions should rather be offered before pregnancy or after birth.

## Conclusions

The costs in pregnant women with obesity who participated in the Mighty Mums intervention were slightly higher than non-participants and a small proportion of the total cost of antenatal and perinatal care. Moreover, the cost per one-kilogram reduction in gestational weight gain was also fairly small, particularly among people who participated actively in the intervention. The results were sensitive to limitations in the retrospective data collection, however, resulting in underestimation of costs for specialized antenatal care, births, and complications, especially among controls. More research is needed to establish the actual value to decision-makers and patients of decreasing gestational weight gain, when such actions would be most beneficial in the long-term, and which interventions are most effective.

## Supplementary Information

**Additional file 1: Figure S1**: Study flow chart. **Table S1**. Descriptive statistics for the ITT and PP study populations. **Table S2**. Average cost (SEK) and gestational weight gain (kg) from ITT and PP analysis, and differences by population groups. **Figure S2**: Distribution of self-reported health status during enrolment and postpartum visit among women participating in the Mighty Mums intervention and controls. Question in the Mighty Mums project relevant for this study.

## Data Availability

The study protocol, statistical analysis plan and informed consent form (all in Swedish) can be requested from the corresponding author. The individual data in this study are not publicly available. Data can only be available after legal review to researchers who meet the criteria for access to this type of sensitive and confidential data (in accordance with the Swedish Ethical Review Act, the Personal Data Act, and the Administrative Procedure Act).

## Abbreviations

BMI: Body mass index
PP: Per protocol
ITT: Intention to treat
SEK: Swedish krona
