# Supplementary material for: Cost effectiveness of a controlled lifestyle intervention for pregnant women with obesity
Source: BMC Pregnancy Childbirth. 2021 Sep 21;21:639. doi: 10.1186/s12884-021-04098-5 (PMC8456662; doi:10.1186/s12884-021-04098-5)
Supplement: Supplementary file 1 — Additional file 1: Figure S1: Study flow chart. Table S1. Descriptive statistics for the ITT and PP study populations. Table S2. Average cost (SEK) and gestational weight gain (kg) from ITT and PP analysis, and differences by population groups. Figure S2: Distribution of self-reported health status during enrolment and postpartum visit among women participating in the Mighty Mums intervention and controls. Question in the Mighty Mums project relevant for this study. [file 12884_2021_4098_MOESM1_ESM.docx]

Supporting information

Cost effectiveness of a controlled lifestyle intervention for pregnant women with obesity

**Authors**

Hanna Gyllensten^1,2,3^, Karin Haby^1,4^, Marie Berg^1,5^, Åsa Premberg^1,4^

Affiliations

^1^ Institute of Health and Care Sciences, University of Gothenburg, Gothenburg, Sweden.

^2^ Centre for Person-Centred Care - GPCC, University of Gothenburg,, Gothenburg, Sweden.

^3^ Department of Clinical Neuroscience, Karolinska Institutet, Stockholm, Sweden.

^4^ Region Västra Götaland, Research and Development Primary Health Care, Gothenburg, Sweden.

^5^ Region Västra Götaland, Sahlgrenska University Hospital, Department of Obstetrics, Gothenburg, Sweden

Corresponding author

Hanna Gyllensten, Associate professor

Postal address: Institute of Health and Care Sciences, University of Gothenburg, Box 457, SE-405 30 Göteborg, Sweden

E-mail: hanna.gyllensten@gu.se

Phone: +46 707 482 412

Content

[Figure S1: Study flow chart. 2](#_Toc82168059)

[Table S1. Descriptive statistics for the ITT and PP study populations. 3](#_Toc82168060)

[Table S2. Average cost (SEK) and gestational weight gain (kg) from ITT and PP analysis, and differences by population groups. 4](#_Toc82168061)

[Figure S2: Distribution of self-reported health status during enrolment and postpartum visit among women participating in the Mighty Mums intervention and controls. 7](#_Toc82168062)

[Question in the Mighty Mums project relevant for this study 8](#_Toc82168063)

Abbreviations: *ITT = intention-t- treat;* *PP = per protocol.*

# Figure S1: Study flow chart.

# Table S1. Descriptive statistics for the ITT and PP study populations.

| Background characteristics | ITT population | |  | PP population | |
| --- | --- | --- | --- | --- | --- |
|  | Intervention n = 434 | Controls n = 867 |  | Intervention n = 115 | Controls n = 841 |
|  | (%) | (%) |  | (%) | (%) |
| Age at enrolment |  |  |  |  |  |
| …≤30 years | 197 (45) | 411 (47) |  | 55 (48) | 404 (48) |
| …>30 years | 237 (55) | 456 (53) |  | 60 (52) | 437 (52) |
| BMI at enrolment |  |  |  |  |  |
| …<30† | **28 (6)** | 5 (1) |  | **6 (5)** | 5 (1) |
| …30–<35 | 270 (62) | **610 (72)** |  | 74 (64) | 595 (71) |
| …35–<40 | 97 (22) | 207 (24) |  | 24 (21) | 201 (24) |
| …≥40 | **39 (9)** | 45 (5) |  | **11 (10)** | 40 (5) |
| Occupation |  |  |  |  |  |
| …paid work | 286 (66) | **674 (78)** |  | 73 (63) | **655 (78)** |
| …other‡ | **148 (34)** | 193 (22) |  | **42 (37)** | 186 (22) |
| Country of birth |  |  |  |  |  |
| …Sweden | 306 (71) | **776 (90)** |  | 81 (70) | **753 (90)** |
| …other | **128 (29)** | 91 (10) |  | **34 (30)** | 88 (10) |
| Use of translator |  |  |  |  |  |
| …yes | **45 (10)** | 17 (2) |  | **14 (12)** | 17 (2) |
| …no | 389 (90) | **850 (98)** |  | 101 (88) | **824 (98)** |
| Nicotine use before pregnancy§ |  |  |  |  |  |
| …yes | 33 (8) | 79 (9) |  | 8 (7) | 77 (9) |
| …no | **401 (92)** | 634 (73) |  | **107 (93)** | 615 (73) |
| Education¶ |  |  |  |  |  |
| …>12 years | 168 (39) | 305 (35) |  | 48 (42) | 298 (35) |
| …≤12 years | 265 (61) | 561 (65) |  | 67 (58) | 542 (64) |
| Mother’s complications |  |  |  |  |  |
| …Gestational diabetes | 15 (3) | 33 (4) |  | 2 (2) | 33 (4) |
| …Gestational hypertension | **11 (3)** | 1 (0) |  | **3 (3)** | 1 (0) |
| …Pre-eclampsia | 32 (7) | 65 (8) |  | 4 (3) | 64 (8) |
| Child’s complications |  |  |  |  |  |
| …Macrosomia | 22 (5) | **77 (9)** |  | 5 (4) | 76 (9) |
| … large for gestational age | 14 (3) | 28 (3) |  | 5 (4) | 28 (3) |
| … small for gestational age | 33 (8) | 46 (5) |  | 10 (9) | 39 (5) |
| …APGAR <7 after 5 min | 9 (2) | 11 (1) |  | 1 (1) | 11 (1) |
| …Preterm birth | 33 (8) | 51 (6) |  | 3 (3) | 44 (5) |

BMI = body mass index; ITT = intention to treat; PP = per protocol; SEK = Swedish krona.

Bold indicates a statistically significant (*P* < 0.05) higher proportion, using z-test to compare between intervention group and controls for the ITT and PP populations. No statistically significant differences were found between the ITT and PP population intervention groups.

Percentages are rounded.

† After transformation to week 15 weights (Johansson et al., 2016)

‡ Including parental leave, unemployment benefits, student loans, and social benefits.

§ Information about nicotine use was missing for 154 controls in the ITT population, of which 149 were in the PP population.

¶ Educational level was missing for 1 intervention participant in the ITT population and 1 control in ITT and PP populations.

# Table S2. Average cost (SEK) and gestational weight gain (kg) from ITT and PP analysis, and differences by population groups.

| Background characteristics | ITT population | | | |  | PP population | | |
| --- | --- | --- | --- | --- | --- | --- | --- | --- |
|  | Intervention | Controls | Difference | |  | Intervention | Controls | Difference |
|  | *Mean* (95% CI) | *Mean* (95% CI) | Unadjusted *M* (95% CI)  Adjusted *M* (95% CI)† | |  | *Mean* (95% CI) | *Mean* (95% CI) | Unadjusted *M* (95% CI)  Adjusted *M* (95% CI)† |
| **Study population** | | | |  | | | | |
|  | **N = 434** | **N = 867** |  | |  | **N = 115** | **N = 841** |  |
| Total cost | 43,181  (40,559 to 45,804) | 33,215 (32,089 to 34,341) | 9,966 (7,103 to 12,829)  9,717 (6,715 to 12,719) | |  | 42,076  (38,062 to 46,091) | 33,124  (32,018 to 34,229) | 8,953 (4,907 to 12,998)  8,655 (4,553 to 12,756) |
| Intervention costs | 6,024  (5,067 to 6,980) | 665  (327 to 1,004) | 5,359 (4,406 to 6,311)  4,892 (3,955 to 5,830) | |  | 6,680  (4,938 to 8,421) | 531  (243 to 819) | 6,149 (4,451 to 7,847)  6,093 (4,448 to 7,758) |
| Gestational weight gain | 10.3  (9.7 to 10.9) | 11.2  (10.8 to 11.7) | −0.9 (−1.7 to −0.2)  −0.2 (−1.0 to 0.6) | |  | 8.9  (7.8 to 10.0) | 11.3  (10.8 to 11.7) | −2.3 (−3.5 to −1.2)  −1.5 (−2.6 to −0.3) |
| **Age at enrolment** | | | |  | | | | |
| **≤30 years** | **n = 197** | **n = 411** |  | |  | **n = 55** | **n = 404** |  |
| Total cost | 41,888  (38,491 to 45,285) | 33,422  (31,883 to 34,961) | 8,466 (4,661 to 12,271)  7,902 (3,756 to 12,048) | |  | 43,896  (36,964 to 50,827) | 33,139  (31,653 to 34,624) | 10,757 (4,220 to 17,295)  10,411 (3,696 to 17,127) |
| Intervention costs | 6,073  (4,599 to 7,546) | 950  (315 to 1,586) | 5,122 (3,455 to 6,790)  4,210 (2,595 to 5,824) | |  | 7,266  (4,444 to 10,088) | 737  (244 to 1,229) | 6,529 (3,742 to 9,317)  6,421 (3,707 to 9,135) |
| Gestational weight gain | 11.3  (10.3 to 12.2) | 11.6  (10.9 to 12.3) | −0.3 (−1.4 to 0.8)  0.9 (−0.3 to 2.1) | |  | 10.1  (8.4 to 11.9) | 11.6  (11.0 to 12.3) | −1.5 (−3.5 to 0.4)  −0.03 (−1.9 to 1.9) |
| **>30 years** | **n = 237** | **n = 456** |  | |  | **n = 60** | **n = 437** |  |
| Total cost | 44,256  (40,382 to 48,131) | 33,028  (31,319 to 34,737) | 11,228 (7,026 to 15,430)  11,230 (7,030 to 15,570) | |  | 40,409  (35,698 to 45,120) | 33,110  (31,424 to 34,795) | 7,299 (2,366 to 12,232)  7,431 (2,417 to 12,446) |
| Intervention costs | 5,983  (4,727 to 7,238) | 408  (106 to 710) | 5,575 (3,374 to 6,776)  5,403 (4,231 to 6,576) | |  | 6,142  (4,260 to 8,024) | 341 (60 to 621) | 5,802 (3,781 to 7,823)  5,853 (3,841 to 7,865) |
| Gestational weight gain | 9.5  (8.8 to 10.2) | 10.9  (10.3 to 11.5) | −1.4 (−2.3 to 0.4)  −1.0 (−2.0 to −0.1) | |  | 7.8  (6.6 to 9.1) | 10.9  (10.2 to 11.6) | −3.1 (−4.5 to −1.6)  −2.7 (-4.1 to −1.3) |
| **BMI at enrolment** | | | |  | | | | |
| **<30** | **n = 28** | **n = 5** |  | |  | **n = 6** | **n = 5** |  |
| Total cost | 45,707  (35,441 to 55,973) | 31,984  (26,379 to 37,589) | 13,723 (1,954–25,493)  13,571 (−2,623 to 29,765)‡ | |  | 32,666  (29,113 to 36,219) | 31,984  (26,379 to 37,589) | 682 (−6,071 to 7,435)  1,198 (−6,118 to 8,514)‡ |
| Intervention cost | 6,240  (3,184 to 9,296) | 7,440  (−184 to 15,064) | −1,200 (−10,169 to 7,770)  −1,138 (−9,467 to 7,190)‡ | |  | 3,671  (−1,237 to 8,578) | *7,440*  (−184 to 15,064) | −3,770 (−13,936 to 6,397)  −3,458 (−11,297 to 4,382)‡ |
| Gestational weight gain | 13.7  (11.9 to 15.5) | 10.6  (3.9 to 17.3) | 3.1 (−4.6 to 10.8)  3.3 (−3.9 to 10.6)‡ | |  | 17.7  (14.6 to 20.7) | 10.6  (3.9 to 17.3) | 7.1 (−0.4 to 14.5)  7.6 (−0.8 to 16.0)‡ |
| **30–<35** | **n = 270** | **n = 610** |  | |  | **n = 74** | **n = 595** |  |
| Total cost | 41,598  (38,331 to 44,866) | 32,069  (30,851 to 33,287) | 9,529 (6,203 to 12,856)  9,100 (5,609 to 12,592) | |  | 43,571  (38,126 to 49,017) | 32,196  (30,969 to 33,424) | 11,375 (5,574 to 17,176)  11,049 (5,391 to 16,706) |
| Intervention costs | 5,818  (4,638 to 6,998) | 427  (118 to 736) | 5,391 (4,206 to 6,576)  4,831 (3,722 to 5,940) | |  | 7,731  (5,445 to 10,017) | 406  (98 to 715) | 7,325 (4,988 to 9,661)  7,179 (4,912 to 9,446) |
| Gestational weight gain | 10.5  (9.9 to 11.2) | 11.9  (11.4 to 12.5) | −1.4 (−2.3 to −0.5)  −1.0 (−1.9 to −0.1) | |  | 8.6  (7.5 to 9.8) | 12.0 (11.4 to 12.5) | −3.3 (−4.6 to −2.1)  −2.7 (−4.1 to −1.4) |
| **35–<40** | **n = 97** | **n = 207** |  | |  | **n = 24** | **n = 201** |  |
| Total cost | 43,670  (39,076 to 48,324) | 35,492  (32,778 to 38,206) | 8,208 (2,961 to 13,454)  8,931 (3,184 to 14,677) | |  | 40,105  (33,866 to 46,344) | 35,319  (32,623 to 38,015) | 4,786 (−2,194 to 11,765)  3,615 (−3,589 to 10,819) |
| Intervention cost | 7,183  (4,913 to 9,453) | 539  (−12 to 1,090) | 6,644 (4,316 to 8,972)  6,513 (4,111 to 8,916) | |  | 5,757  (2,362 to 9,153) | 463  (−43 to 968) | 5,295 (1,873 to 8,717)  6,078 (2,498 to 9,658) |
| Gestational weight gain | 9.6  (8.3 to 11.0) | 10.0  (9.1 to 10.9) | −0.4 (−2.1 to 1.3)  0.5 (−1.3 to 2.3) | |  | 8.4  (5.9 to 10.9) | 10.0  (9.1 to 10.9) | −1.6 (-4.5 to 1.3)  −0.4 (-3.6 to 2.8) |
| **≥40** | **n = 39** | **n = 45** |  | |  | **n = 11** | **n = 40** |  |
| Total cost | 51,036  (38,553 to 63,520) | 38,408  (31,963 to 44,854) | 12,628 (−2,625 to 27,881)  16,840 (−1,085 to 34,765)‡ | |  | 41,455  (30,832 to 52,078) | 36,025  (30,015 to 42,034) | 5,430 (−7,394 to 18,254)  9,237 (−1,749 to 20,223)‡ |
| Intervention costs | 4,408  (1,836 to 6,980) | 3,720  (−179 to 7,619) | 688 (−4,093 to 5,469)  193 (−4,171 to 4,557)‡ | |  | 3,262  (−729 to 7,254) | 1,860  (−879 to 4,599) | 1,402 (−3,774 to 6,579)  2,396 (−2,417 to 7,210)‡ |
| Gestational weight gain | 8.3  (6.2 to 10.3) | 7.1  (5.2 to 9.0) | 1.2 (−1.6 to 4.0)  1.6 (−1.6 to 4.8)‡ | |  | 7.5  (2.6 to 12.3) | 7.3  (5.3 to 9.4) | 0.1 (−5.5 to 5.7)  −0.1 (−6.4 to 6.2)‡ |
| **Occupation** | | | |  | | | | |
| **paid work** | **n = 286** | **n = 674** |  | |  | **n = 73** | **n = 655** |  |
| Total cost | 46,090  (42,366 to 49,814) | 34,113  (32,788 to 35,438) | 11,977 (8,273 to 15,681)  11,407 (7,694 to 15,121)‡ | |  | 45,407  (39,944 to 50,869) | 33,923  (32,553 to 35,292) | 11,484 (6,151 to 16,816)  11,113 (5,927 to 16,300)‡ |
| Intervention costs | 5,653  (4,572 to 6,733) | 828  (405 to 1,251) | 4,825 (3,694 to 5,956)  4,693 (3,578 to 5,809)‡ | |  | 7,143  (5277 to 9,009) | 653  (272 to 1,034) | 6,490 (4,637 to 8,342)  6,482 (4,633 to 8,332)‡ |
| Gestational weight gain | 11.0  (10.3 to 11.7) | 11.5  (11.0 to 12.0) | −0.5 (−1.4 to 0.4)  −0.2 (−1.1 to 0.6)‡ | |  | 9.9  (8.5 to 11.2) | 11.5  (11.0 to 12.0) | −1.6 (−3.1 to −0.1)  −1.5 (−3.0 to 0.03)‡ |
| **other** | **n = 148** | **n = 193** |  | |  | **n = 42** | **n = 186** |  |
| Total cost | 37,560 (34,700 to 40,423) | 30,079  (28,217 to 31,941) | 7,481 (3,979 to 10,984)  5,432 (1,867 to 8,998) | |  | 36,289  (31,705 to 40,872) | 30,309  (28,363 to 32,256) | 5,979 (676 to 11,282)  3,899 (−1,832 to 9,630) |
| Intervention costs | 6,740  (4,978 to 8,503) | 96  (−90 to 283) | 6,644 (4,797 to 8,490)  5,380 (3,608 to 7,152) | |  | 5,875  (2,572 to 9,177) | 100  (−100 to 300) | 5,775 (2,456 to 9,094)  5,239 (2,125 to 8,352) |
| Gestational weight gain | 9.0  (8.1 to 9.8) | 10.2  (9.2 to 11.2) | −1.3 (−2.6 to 0.1)  −0.2 (−1.6 to 1.2) | |  | 7.3  (5.6 to 9.0) | 10.4  (93 to 11.4) | −3.1 (−5.1 to −1.1)  −1.7 (−4.0 to 0.6) |
| **Nicotine use before pregnancy** | | | |  | | | | |
| **yes** | **n = 33** | **n = 79** |  | |  | **n = 8** | **n = 77** |  |
| Total cost | 35,055  (28,295 to 41,815) | 33,445  (29,812 to 37,077) | 1,610 (−6,164 to 9,384)  2,077 (−6,122 to 10,276)‡ | |  | 31,744  (21,067 to 42,420) | 33,829  (30,319 to 37,339) | −2,085 (−14,430 to 10,259)  165 (−12,947 to 13,277)‡ |
| Intervention costs | 2,865  (821 to 4,910) | 235  (−198 to 669) | 2,630 (601 to 4,659)  2,378 (466 to 4,291)‡ | |  | 3,351  (−763 to 7,465) | 242  (−207 to 690) | 3,109 (−1,574 to 7,792)  3,399 (−1,424 to 8,222)‡ |
| Gestational weight gain | 10.8  (8.1 to 13.6) | 9.7  (8.0 to 11.3) | 1.2 (−2.1 to 4.4)  1.0 (−1.9 to 4.0)‡ | |  | 5.9  (1.6 to 10.2) | 9.6  (7.9 to 11.4) | −3.8 (−8.8 to 1.2)  −4.0 (−8.3 to 0.4)‡ |
| **no** | **n = 401** | **n = 634** |  | |  | **n = 107** | **n = 615** |  |
| Total cost | 43,850  (40,974 to 46,726) | 33,668  (32,293 to 35,042) | 10,182 (7,046 to 13,319)  10,037 (6,696 to 13,377) | |  | 42,849  (38,796 to 46,902) | 33,450  (32,088 to 34,812) | 9,399 (5,155 to 13,643)  9,056 (4,703 to 13,409) |
| Intervention costs | 6,284  (5,324 to 7,243) | 880  (422 to 1,338) | 5,403 (4,310 to 6,497)  4,904 (3,850 to5,958) | |  | 6,929  (5,217 to 8,640) | 696  (287 to 1,104) | 6,233 (4,458 to 8,108)  6,155 (4,327 to 7,983) |
| Gestational weight gain | 10.3  (9.7 to 10.9) | 11.4  (10.8 to 11.9) | −1.1 (−1.8 to −0.3)  −0.3 (−1.1 to 0.5) | |  | 9.2  (8.1 to 10.3) | 11.4  (10.8 to 11.9) | −2.2 (−3.5 to −1.0)  −1.2 (−2.5 to 0.1) |

BMI = body mass index; CI = confidence interval; ITT = intention to treat; PP = per protocol; SEK = Swedish krona

Figures are rounded.

† Adjusted for country of birth, use of interpreter services, occupation (not adjusted for in the comparison by occupation), and BMI at enrolment (not adjusted for in the analysis by BMI at enrolment).

‡ Bootstrapped results sensitive to small samples, thus results were calculated based on fewer complete replications (ITT: BMI<30: 625 replications; BMI≥40: 876 replications; Nicotine use adjusted samples: 636 replications; PP: BMI<30: 830-999 replications; paid work: 995 replications, BMI≥40: 981-995 replications; Nicotine use: 616 replications).

*.*


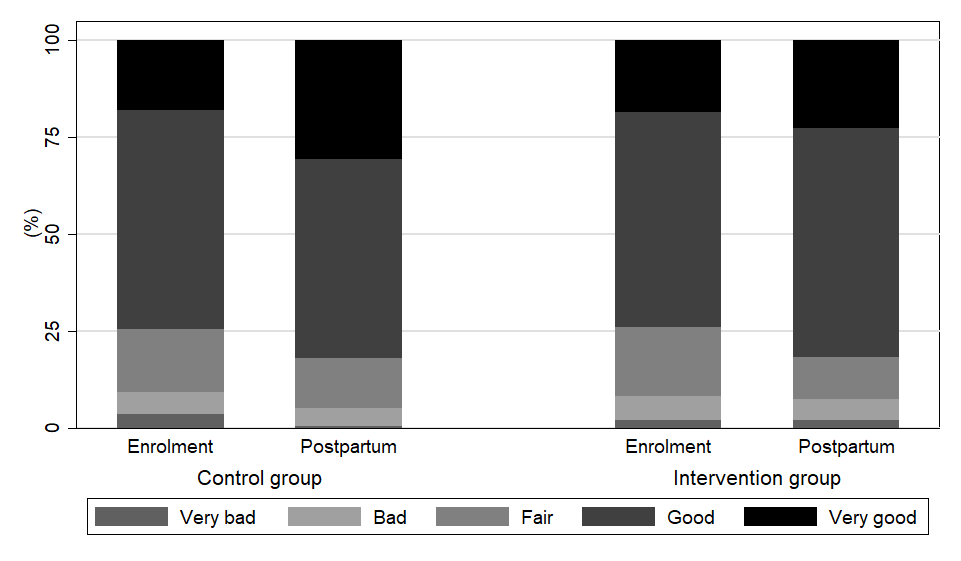


# Figure S2: Distribution of self-reported health status during enrolment and postpartum visit among women participating in the Mighty Mums intervention and controls.

# Question in the Mighty Mums project relevant for this study

How do you perceive your general health status during the three months before you got pregnant?

Very good Good Neither good or bad Bad Very bad Do not know
